# Toward *Operando* Structural, Chemical, and Electrochemical Analyses of Solid-State Batteries Using Correlative Secondary Ion Mass Spectrometry Imaging

**DOI:** 10.1021/acs.analchem.3c01059

**Published:** 2023-06-22

**Authors:** Luca Cressa, Yanyan Sun, Dustin Andersen, Mathieu Gerard, Olivier De Castro, Dennis Kopljar, Maryam Nojabaee, Kaspar Andreas Friedrich, Guido Schmitz, Tom Wirtz, Santhana Eswara

**Affiliations:** † Advanced Instrumentation for Nano-Analytics (AINA), Luxembourg Institute of Science and Technology, 41 Rue du Brill, Belvaux L-4422, Luxembourg; ‡ Chair of Materials Physics, Institute for Materials Science, University of Stuttgart, Heisenbergstr. 3, Stuttgart 70569, Germany; § German Aerospace Center (DLR), Institute of Engineering Thermodynamics, Pfaffenwaldring 38-40, Stuttgart 70569, Germany; ∥ Institute of Building Energetics, Thermal Engineering and Energy Storage (IGTE), University of Stuttgart, Pfaffenwaldring 6, Stuttgart 70569, Germany

## Abstract

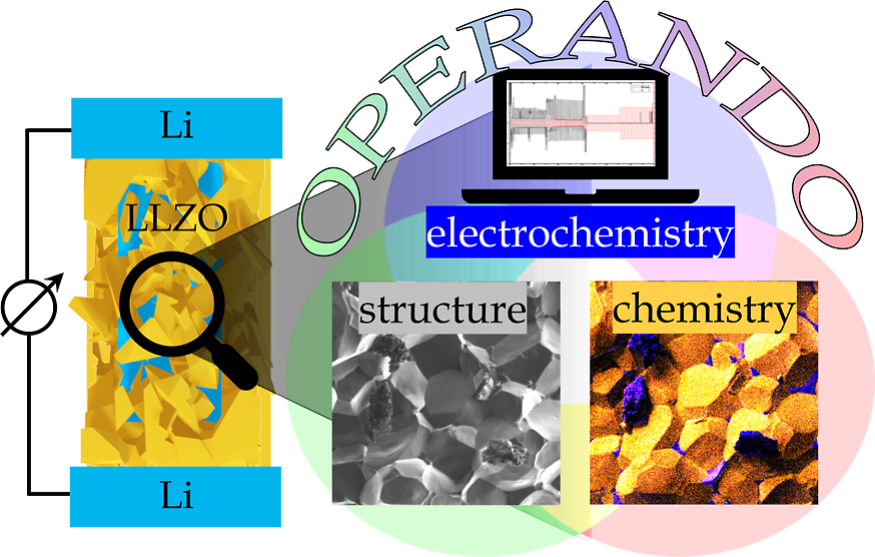

The global transition from fossil fuels to green energy underpins the need for efficient and reliable energy storage systems. Advanced analysis and characterization of battery materials is not only important to understand fundamental battery properties but also crucial for their continued development. A deep understanding of these systems is often difficult to obtain through only pre- and/or post-mortem analyses, with the full complexity of a battery being hidden in its operational state. Thus, we have developed an *operando* methodology to analyze solid-state batteries (SSBs) structurally as well as chemically before, during, and after cycling. The approach is based on a specially designed sample holder, which enables a variety of electrochemical experiments. Since the entire workflow is performed within a single focused ion beam scanning electron microscope equipped with an in-house developed magnetic sector secondary ion mass spectrometer, we are able to pause the cycling at any time, perform analysis, and then continue cycling. Microstructural analysis is performed via secondary electron imaging, and the chemical mapping is performed using the secondary ion mass spectrometer. In this proof-of-concept study, we were able to identify dendrites in a short-circuited symmetric cell and to chemically map dendritic structures. While this methodology focuses on SSBs, the approach can directly be adapted to different battery systems and beyond. Our technique clearly has an advantage over many alternatives for battery analysis as no transfer of samples between instruments is needed and a correlation between the microstructure, chemical composition, and electrochemical performance is obtained directly.

The energy transition from fossil fuels to sustainable and green energy requires not only advances in energy harvesting technologies but also urgent breakthroughs in the field of energy storage systems. The research interest is enormous, and many different battery technologies are under investigation;^1−6^ nonetheless, only limited options for advanced analysis and characterization of novel battery materials are available. Meanwhile, the battery community agrees that pre- and post-mortem analyses are often insufficient to fully understand what is occurring inside of batteries during operation, and *operando* approaches are required to overcome this challenge.^7,8^ Several studies, for instance,^9−16^ have been carried out, and researchers have developed innovative setups and workflows for in situ and/or *operando* studies of batteries. While Masuda et al.^10^ paved the way toward in situ time-of-flight secondary ion mass spectrometry (TOF-SIMS) imaging for solid-state batteries (SSBs), they cannot exclude interference between cycling and analysis. Mathayan et al.^11,12^ performed *operando* elastic recoil detection analysis and Rutherford backscattering spectrometry to study lithium and oxygen transport, but their methodology is limited to ultrathin batteries with a maximum thickness of a few μm, and more importantly microstructural changes are not captured. Otoyama et al.^14^ observed structural changes in SSBs via *operando* confocal microscopy in an innovative approach; however, this work is constrained by the spatial resolution of the optical microscope and lacks a chemical characterization method. Yamagishi et al.^16^ developed a very promising *operando* TOF-SIMS methodology for studying SSBs that could observe the dynamic evolution of the distribution of Li in the graphite anode during operation. Their study was on a rather large field of view (>100 μm) and focused on the Li distribution on the anodic side. The TOF principle inevitably involves pulsing of the primary (or secondary) ion beam, resulting in an associated duty cycle. Hence, image acquisition times (for a given number of secondary ions per pixel) are orders of magnitude longer in comparison to those of magnetic sector SIMS imaging, which works with a direct current (DC) beam and no duty cycle. Furthermore, the higher extraction efficiency and higher overall transmission offered by magnetic sector SIMS result in superior detection limits when compared to those of TOF-SIMS. In this work, we present a methodology for performing *operando* magnetic sector SIMS with a lateral resolution for chemical imaging of 15 nm and a depth resolution of ∼4 nm, with a focus on the degradation mechanisms inside solid electrolytes.

The limited availability of advanced *operando* analysis methodologies for the analysis of lithium ion batteries is a consequence of the following three factors: (1) implementation of *operando* modalities within analytical instruments often requires major instrumental modifications; (2) the air and moisture sensitivity of many battery components necessitates a constant inert gas atmosphere (or vacuum); (3) most conventional chemical analysis techniques (energy-dispersive X-ray spectroscopy and Raman spectroscopy) are not suitable for analyzing low-Z elements, such as hydrogen (H) or lithium (Li), which are essential when investigating batteries.

In the present article, it is elucidated how these challenges have been addressed and overcome. The undertaken approach is based on a custom-designed sample holder which has been adapted to a focused ion beam scanning electron microscopy (FIB-SEM) instrument. With the herein introduced methodology and with the example of solid-state half-cell samples, the feasibility of a correlative structural, chemical, and electrochemical workflow within FIB-SEM-SIMS^17^ is demonstrated. We believe that the approach we present provides an unprecedented analysis for tackling unsolved questions such as Li-electroplating in SSBs or transition-metal diffusion to accelerate battery research.

## Design of the *Operando* Sample Holder

**Figure 1:**
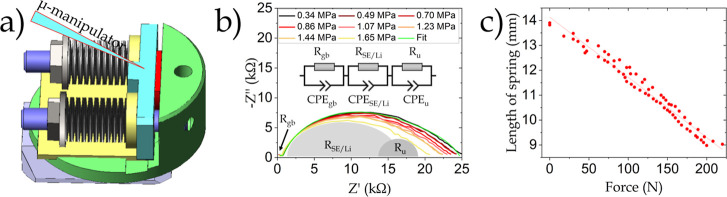
(a) Schematics of the custom-designed sample holder; the red component represents the semi-circular sample clamped between two electrically insulated plates (green and cyan). (b) Example of interface resistance reduction with increasing pressure. The applied pressure from 0.34 to 1.65 MPa shows a considerable reduction of the interface impedance *R*_SE/Li_. The equivalent circuit for the fitting (0.34 MPa) is shown as an inset, and the impedance contributions of the grain boundary (*R*_gb_), solid electrolyte with the Li electrodes (*R*_SE/Li_), and an additional resistance (*R*_u_) are exemplarily shown. The fitting parameters are given in Table S1. (c) Calibration curve of the sample holder correlating “length of the spring” to “applied force”.

To our knowledge, there is no existing commercial solution for *operando*-correlative SIMS analysis of SSBs. Therefore, a custom-designed sample holder was developed. The design fulfils the following technical requirements: (1) possibility to clamp a sample between two plates which are electrically conductive but separated by an insulator; (2) possibility to modify and control the force which is applied to the sample between the two plates; (3) sufficiently small to fit within the inert gas transfer system (IGTS) compatible with the airlock system of the FIB-SEM instrument (e.g. dimensions, sample stage adapter, materials). A schematic of the sample holder is shown in Figure 1a. Furthermore, a custom-made IGTS (developed together with Ferrovac AG^18^ see Supporting Information Figure S4) permits a contamination-free sample transfer from glove box to instrument and vice versa. The FIB-SEM instrument is equipped with an in-house-designed magnetic sector SIMS, and details about the FIB-SEM-SIMS instrument and its performance can be found elsewhere.^17^ The implementation of SIMS in the present *operando* study presents a huge advantage in terms of chemical analysis,^19^ allowing to detect and measure the distribution of low-Z elements such as H^20^ and Li.^21,22^ Li often plays an important role in degradation mechanisms due to its high ionic mobility and capability to form dendrites,^23−28^ and therefore, it is essential to observe the evolution of the Li distribution.

### Electrochemical Experiments

Electrochemical experiments such as constant current (CC) cycling or electrochemical impedance spectroscopy (EIS) were performed using a SP-150 potentiostat from BioLogic. Preliminary tests (CC dis/charge) with commercial batteries as well as resistance measurements with surface-mount resistors can be found in the Supporting Information under “Electrochemical Experiments”. The EIS measurements (Figure 1b) were done in potentiostatic mode in a frequency range from 1 MHz to 1 Hz with an amplitude of 5 mV. The large semi-circle, which gets smaller with increased pressure, was identified as the solid electrolyte-lithium *R*_SE/Li_ interface impedance.

The measurements in Figure 1b,c as well as all tests were performed with commercial coin cells (Supporting Information) and performed outside the FIB-SEM instrument but in the controlled atmosphere of a glovebox. Additional EIS tests comparing the impedance of the same sample in a conventional Swagelok cell and in the *operando* holder are in the Supporting Information (Figure S2d).

### Pressure Control

The springs in the sample holder are composed of multiple BelleVille washers, which are capable of exerting a force of 20–220 N. The springs were calibrated with force sensors from SingleTact (https://www.singletact.com/) and Figure 1c shows the calibration curve of the “applied force” vs “length of the spring”. This feature is relevant for multiple reasons. First, the effective contact area between Li and the solid electrolyte can be enhanced by increasing the pressure, resulting in decreased interfacial impedance. Further, SSBs under high pressure present longer lifetimes and are under investigation for potential application.^29−33^ An experimental example of the impedance-pressure dependence can be seen in Figure 1b, where a decrease in the solid-electrolyte-lithium interphase resistance can be observed with increasing pressures from 0.34 to 1.65 MPa.

### Adaptation to the Inert Gas Transfer System and Microscope

The sample holder dimensions (diameter = 3.2 cm and height = 1.9 cm) and the materials (stainless steel and Al_2_O_3_) were carefully selected to ensure full compatibility with the operation of the IGTS as well as with the FIB-SEM instrument. Electrochemical analyses inside the FIB-SEM instrument can be performed by connecting one electrode of the potentiostat to the sample stage bias connection of the microscope and the second electrode to the micro-manipulator (i.e., a microscopic needle inside the FIB-SEM, movable along the three spatial axes, typically used for FIB lamella preparation for transmission electron microscopy, see schematics in Figure 1 and the SEM image in Figure S2b).

### Final Experimental Configuration

**Figure 2:**
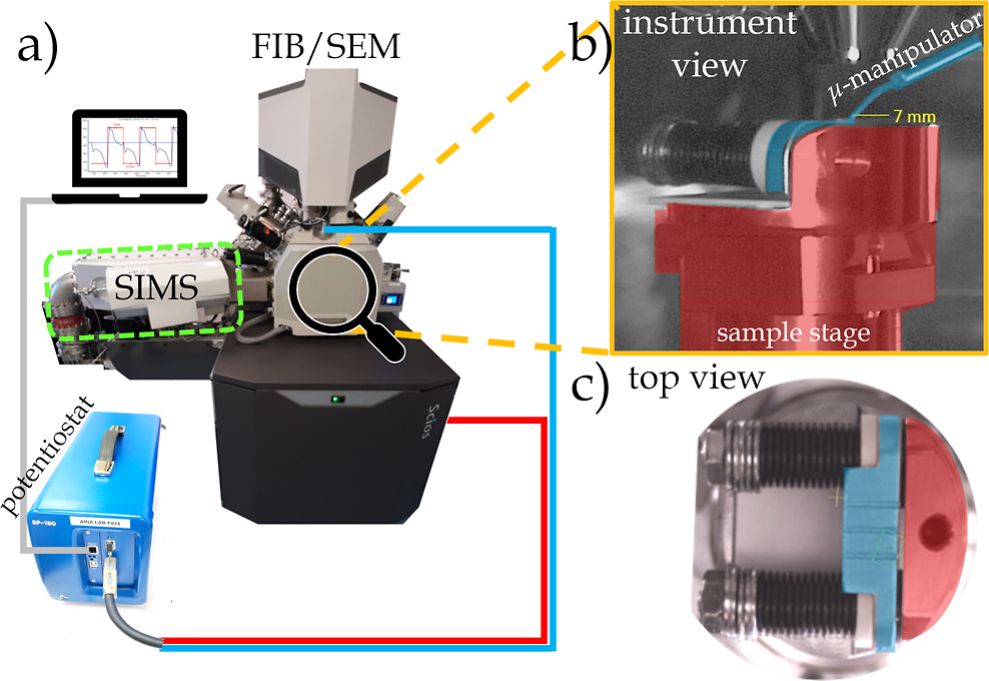
(a) Schematic of the instrumental set-up of the FIB-SEM instrument and the SIMS add-on (green box). The potentiostat (BioLogic SP-150) is connected to different parts of the instrument: via a red cable to the sample stage bias connection and via a blue cable to the micro-manipulator. The gray cable connects to the PC and is responsible for monitoring and controlling the electrochemical experiments. b) Zoom-in view inside the instrument when the sample holder is introduced. The sample stage plus the part of the sample holder which is electrically connected are highlighted in red and indicate one polarity. The FIB micro-manipulator and the electrically connected plate are highlighted in blue and indicate the other polarity. (c) Top view of the sample holder; the slit between the red and blue plates is where the sample is clamped. The sample holder contains parts made of aluminum oxide Al_2_O_3_ to ensure electrical insulation between the two terminals.

The final experimental configuration (see Figure 2a) looks as follows: on the FIB-SEM instrument, an external potentiostat is connected via two electrodes. One electrode (blue cable in Figure 2a) is attached to the micromanipulator, which can be moved along the three spatial axes inside the instrument and can contact the electrode plate of the *operando* sample holder (blue highlighted in Figure 2b,c). The second electrode (red cable) of the potentiostat is connected to the sample stage bias connection, which is electrically isolated from the rest of the microscope and is highlighted in red in Figure 2b,c. A third cable (grey) connects the potentiostat to a PC, which monitors and controls the electrochemical experiments. The dashed green box in Figure 2a indicates the SIMS add-on. To switch between “electrochemical analysis” and “SEM/SIMS analysis” modes, the micromanipulator is retracted, and the secondary ion extraction box is inserted by means of automated piezo-positioners between the sample and the nozzle of the ion column (details described by De Castro et al.^17^).

## Materials and Methods

For this proof of concept, Li_7_La_3_Zr_2_O_12_ (LLZO) powder from Jining CreaTech Energy Technology Co., Ltd, China, was used to produce the pellets. The LLZO pellets in this work were prepared using similar procedures as Zhang et al.,^34^ Jiang et al.,^35^ and Ganesh Kumar et al.,^36^ resulting in high-density pellets (5.1 g/cm^3^). After sintering, the pellets were polished using different sandpapers (grit number #4000) and have final dimensions of 11 mm in diameter and 1–1.5 mm in thickness. The metallic Li-foil which is used to assemble the half-cell is from Alfa Aesar (CAS-No. 7439-93-2). No liquid electrolyte or special pre-treatment of the Li foils was used in this work.

**Figure 3:**
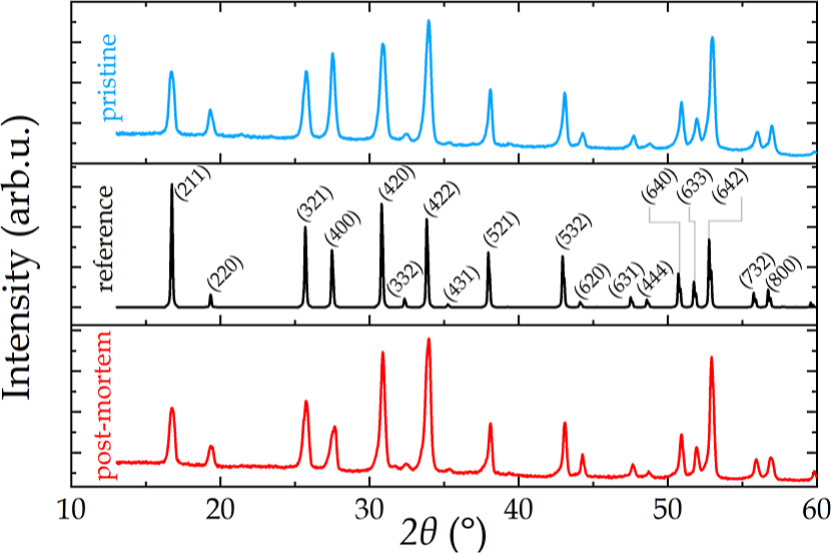
XRD patterns of a pristine LLZO pellet (blue), a reference pattern (black) for cubic structure taken from the PDF 04-022-7984 database, and a post-mortem sample (red).

Figure 3 shows the phase purity of a pristine LLZO pellet (blue), determined by X-ray diffraction (XRD, Bruker AXS D8 Discover) using the Cu-Kα1 radiation with a 2Θ step size of 0.02° (scan rate = 0.02°/180 s). A reference pattern (black) for cubic structure was taken from the PDF 04-022-7984 database, and a post-mortem (red) XRD pattern is plotted as well. The latter shows no additional peaks, indicating that either no phase change occurred during cycling or that the volume fraction of any new phases is too low to be detected by XRD.

### Sample Preparation

SIMS analysis sets two major sample requirements: (1) for a good secondary ion extraction, a homogenous electrical field needs to be generated between the sample and the extraction box. This is only possible when analyzing planar samples. (2) The regions of interest (ROIs), in this case degradation artifacts related to cycling, should be directly accessible for analysis. According to the latter requirements, the ROIs should be on an exposed surface or within a depth that can be reached by FIB-milling. These factors brought us to the conclusion that the use of a regular circular pellet (circular cell) is not appropriate for our *operando* approach. As the curvature of a circular cell is unfavorable, especially for SIMS, a compromise must be found; hence, we decided to aim for the analysis of a semi-circular solid-state half-cell.

The LLZO pellet was split into two parts to obtain a semi-circular pellet with one straight side. The semi-circular LLZO pellet was assembled between two semi-circular Li-foils, forming a Li/LLZO/Li sandwich, which was then mounted in the custom-designed sample holder. The straight and planar side is mounted in such a way on the sample holder that it faces the electron-/ion-beam. One drawback is that the system now operates with unconventional dimensions for a solid-state half-cell, making the electrochemical cycling not directly comparable to commercial cyclers. Three different ways to cut and treat the surface of interest are reported in the Supporting Information under “1.1 Sample Preparation”. In this proof of concept, however, we proceed with the most promising option of the three.

### SIMS Analysis

SIMS imaging was performed using a Thermo Fisher Scios DualBeam FIB-SEM equipped with an in-house developed double-focusing magnetic sector SIMS system, which allows the detection of multiple masses in parallel.^17,19^ The FIB consists of a gallium liquid metal ion source producing ^69^Ga^+^ primary ions. The secondary ions that were collected and imaged are: ^7^Li^+^, ^139^La^+^, and ^155^LaO^+^. The SIMS measurements were carried out with a primary ion beam energy of 30 keV, a beam current of 0.1 and 0.3 nA, and dwell times between 0.5 and 1 ms. The images were recorded with a resolution of 512 × 512 pixels and fields of view between 20 × 20 and 30 × 30 μm. To extract positive secondary ions, the sample was biased to +500 V, which resulted in a primary ion impact energy of 29.5 keV. Data analysis was performed using the free software ImageJ^37^ and the commercial software AVIZO (Version 2021.1., Thermo Fisher).

The sample was introduced inside the transfer chamber for vacuum drying (∼1 h) and subsequently inside an Ar-glove box ([O_2_] = ∼0.6 ppm; [H_2_O] = ∼0.8 ppm) for the assembly of the half-cell. The force with which the sample was clamped in between the two plates of the sample holder was approximately 76 N, resulting in a pressure of ∼1.93 MPa. The value for the force was obtained via the calibration curve shown in Figure 1c, and the pressure was calculated by considering the effective surface area on which this force was applied.

After preparing and properly mounting the sample into the sample holder, the latter was placed inside a transfer shuttle (see Figure S4), which is air-tight and allows contamination-free transfer from the glove box to FIB-SEM and vice versa. Once the sample holder was introduced inside the FIB-SEM instrument (see Figure 2), the sample was structurally as well as chemically analyzed.

## Results and Discussion

### Proof of Concept

**Figure 4:**
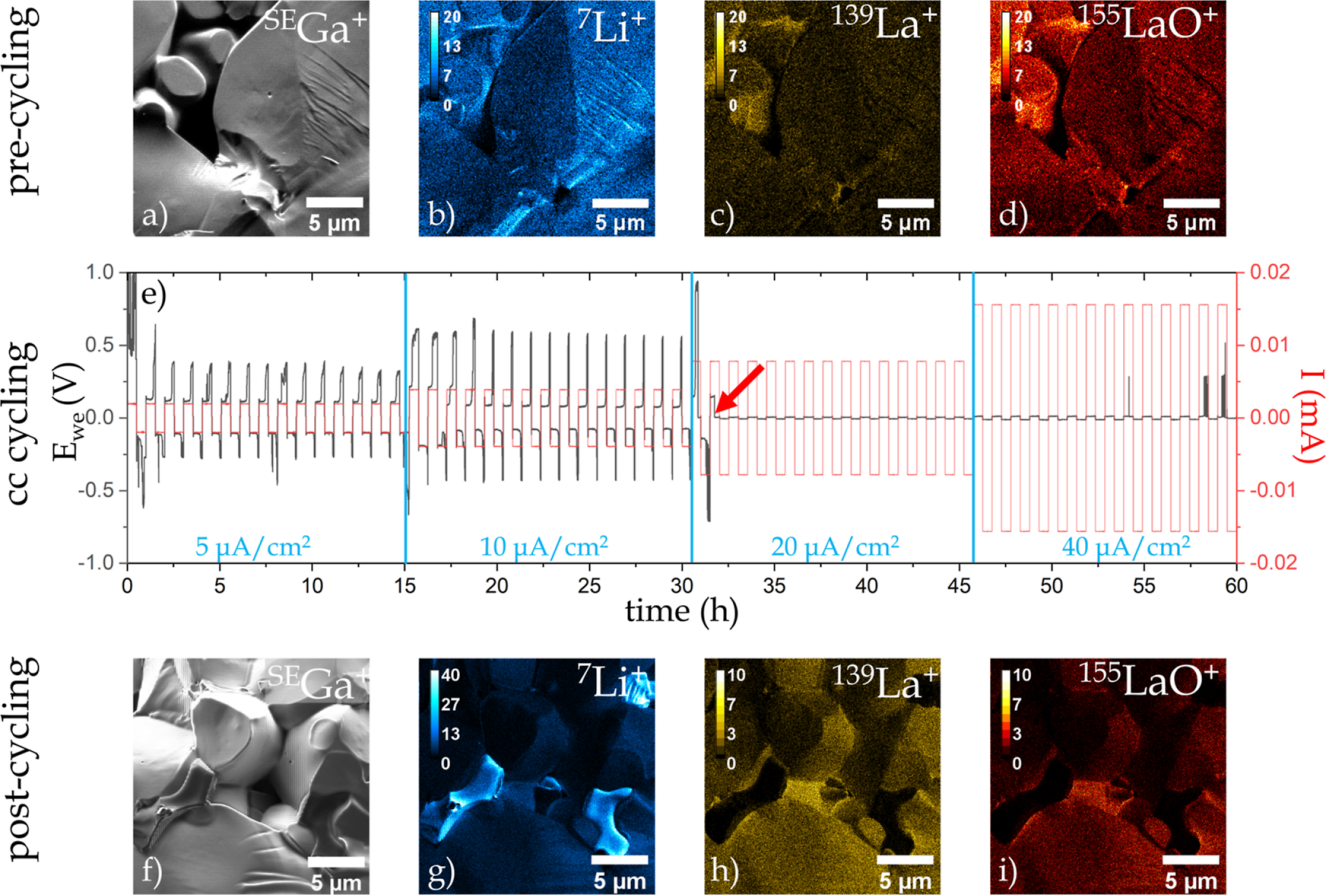
Correlative SIMS analysis in pre-cycled (a–d) and in post-cycled state (f–i). (a,f) Secondary electron image generated by ^69^Ga^+^ irradiation (*E* = 30 keV, *I* = 0.1 nA, and *t*_dwell_ = 2–3 μs). (b–d,g–i) show elemental maps of ^7^Li^+^, ^139^La^+^, and ^155^LaO^+^ for both states. (e) Current and voltage profiles for the 60 cycles = 15 cycles × 4 different current densities (5, 10, 20, and 40 μΑ/cm^2^). During the 2nd cycle of the 20 μΑ/cm^2^ current density, the sample short-circuited (red arrow), as indicated by the voltage profile, which drops close to zero.

The structural analysis was done via FIB- secondary electron (SE) imaging and elucidated the granular structure of the pellet, including porosities (Figure 4a). X-ray computer tomography measurements on other samples synthesized using a similar procedure revealed a porosity below 8%. In the pre-cycling analysis, only a single phase is identifiable based on its structure and chemical composition. The XRD pattern in Figure 3 shows no additional peak when comparing the pristine LLZO pellet and the reference pattern (PDF 04-022-7984 database), indicating the presence of one single phase. SIMS analysis confirms that the only possible composition is LLZO (Figure 4b,c,d), showing the chemical images of ^7^Li^+^, ^139^La^+^, and ^155^LaO^+^, respectively. The fact that no additional phase can be seen in the XRD data nor in the secondary electron images and the homogeneous distribution of the detected secondary ions are strong indicators that the sample preparation as well as the sample transfer have been contamination-free.

The slight difference of the SE as well as secondary ion signals on different faces of the particles is due to topographic effects. The variations of the detected secondary ion intensities within one single phase are in accordance with Sigmund’s theory of sputtering^38^ as well as many studies on particle-matter interactions.^39−42^ We observe in Figure 4b,c,d,g,h,i a typical case of differential sputter yields related to different local incidence angles of the primary beam. Due to the intrinsic granular shape of LLZO particles and the fact that the sample has not been polished, the exposed surface of the pellet shows significant topography (up to μm-scale see Supporting Information under “1.1 Sample Preparation”). When the primary beam hits surfaces with different inclinations, a variation of the sputter yield and hence secondary ion intensities can be observed. Typically, the sputter yield increases up to the glancing angle, and beyond that, the sputter yield decreases due to reflection of the primary ions.^43^ Consequently, it is impossible to obtain SIMS data with perfect homogenous extraction and detection on relatively rough surfaces. However, the relative ratios between the sputter yields of the elements are unaffected by the surface topography.

Right after the pre-cycling analysis, constant current cycling was performed with four different settings: 5, 10, 20, and 40 μΑ/cm^2^ and 15 cycles for each (see Figure 3e). One full cycle (“charging and discharging”) lasts 1 h, resulting in a total duration of 60 h for the electrochemical experiment. During the first 15 cycles at 5 μΑ/cm^2^, we can see that the voltage is between −0.5 and 0.5 V; however, the voltage profile is not totally symmetric, and it shows a slight shift toward positive voltages. Cycles 16–30 were performed with 10 μΑ/cm^2^, resulting in a voltage profile between −0.7 and 0.7 V and a more significant shift toward positive voltages. Note that the voltage needed to maintain the current drops at the 18th cycle (3rd with a current density of 10 μΑ/cm^2^), indicating a drop in resistance, which could be attributed to the nucleation and growth of dendrites. During cycle 32, (2nd cycle with a current density of 20 μΑ/cm^2^) a sudden drop in polarization voltage to approximately 0 V occurred, indicating a short-circuit failure of the cell. The fact that, at this point, essentially no potential difference between both electrodes was measured when maintaining a CC means that the electrical conductivity in the LLZO pellet must have abruptly increased during the cycling. This abrupt change of the electrical properties could result from a compositional change linked to a sudden percolation transition switching to an intrinsically higher electrical conductivity than LLZO.

After this cycling, the sample can be directly analyzed on the same instrument without the need of transfer. The structural analysis revealed the presence of a second darker phase at LLZO grain boundaries and in intergranular cavities (see Figure 4f). Subsequent SIMS analysis shows a significant increase in counts for the ^7^Li^+^ signal at the locations of the dark phase (Figure 4g), while the intensities of the ^139^La^+^ and ^155^LaO^+^ signals (Figure 4h,i) show no signal at all on the dark phase, a clear indication that a new almost pure Li phase has formed. XRD measurements on a post-mortem LLZO sample (Figure 3) reveal no additional peak when compared to the pristine pellet. The latter indicates that the volume fraction of the new phase might be below the detection limit of the technique. This further underlines the necessity for direct chemical imaging. This need is fulfilled by nanoscale SIMS imaging, which allows chemical mapping with a lateral resolution of 15 nm and a depth resolution of ∼4 nm.^17^ The pre- and post-cycling ROIs do not represent the exact same location, as it is not possible to predict where degradations and potential Li-dendrites will emerge.

**Figure 5:**
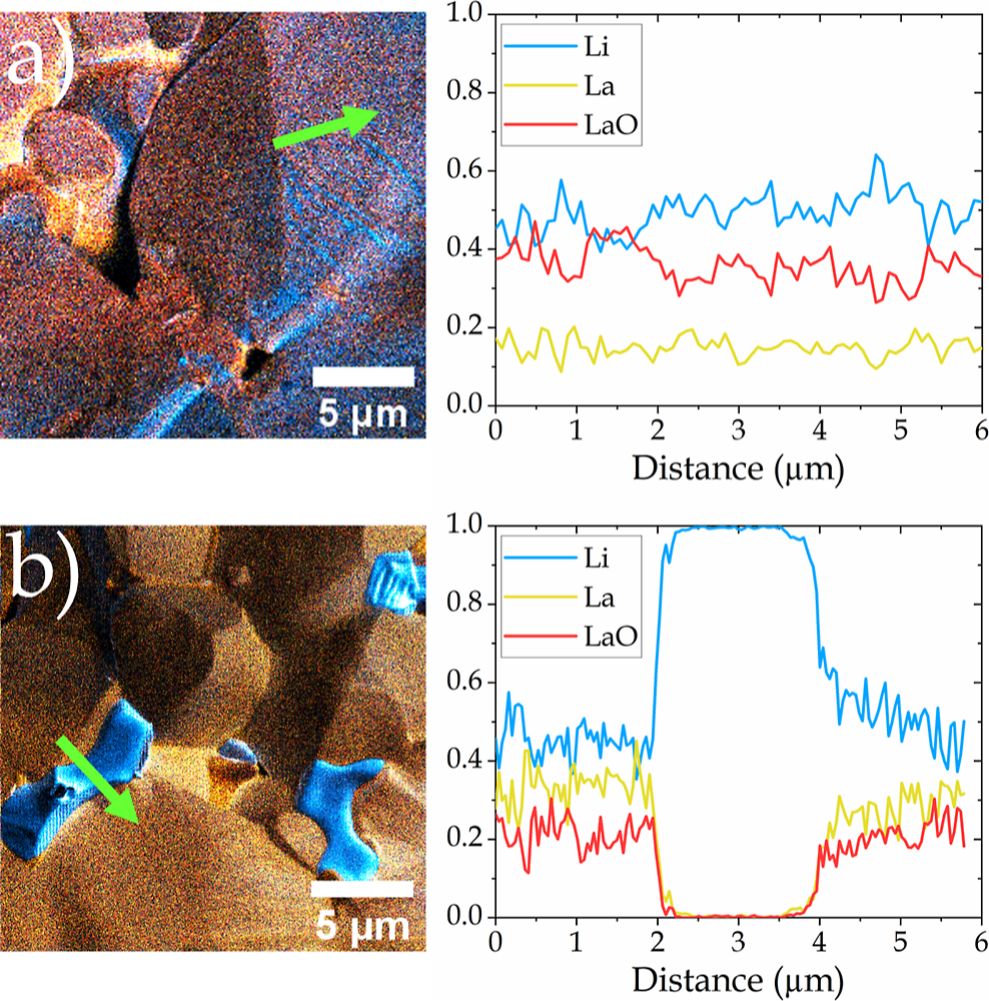
Merged elemental maps (from Figure 4) of ^7^Li^+^ (blue), ^139^La^+^ (yellow), and ^155^LaO^+^ (red) and corresponding line profiles of the regions indicated by the green arrow for the (a) pre-cycling and (b) post-cycled states. The counts for the line profile diagrams have been normalized for a qualitative comparison.

Figure 5a,b show overlays of the three elemental maps of ^7^Li^+^, ^139^La^+^, and ^155^LaO^+^ of the pre- and post-cycled states (from Figure 4), respectively. The pre-cycled state (Figure 5a) shows that the chemical distribution is mostly homogeneous for all detected elements. The signals have been normalized for a qualitative comparison of the line profiles. The latter (Figure 5b) confirms the hypothesis of a new phase, elucidating a strong increase of the ^7^Li^+^ signal and the absence of ^139^La^+^ and ^155^LaO^+^. A new phase has been created as a consequence of cycling based on the experimental observations. This new phase contains Li and no detectable La or LaO, and it grows mainly at grain boundaries and intergranular cavities. The formation as well as a possible growth of this phase might affect the electrochemical properties and could be responsible for the short-circuit (see Figure 4e).

By comparing the SE images in Figure 4a,f, it becomes obvious that a darker phase appeared in post-cycled analysis. Typically, we would expect higher SE as well as higher secondary ion signals due to a high impact angle and thereby increased sputter yields. However, the dark areas in the SE image appear very bright in the chemical map of ^7^Li^+^, hence indicating a high Li concentration and not a topographic effect.

This proves the identification of the newly visible structures as a distinct phase indicative of a dendrite and not just an artifact due to sample topography. The latter is further supported by the fact that the relative ratios of the different elements within this structure are very different from those within the LLZO grains.

While several researchers were able to observe similar^23,25,26,44^ degradation products after short-circuiting of LLZO-based batteries, in this study, it was possible to directly map Li on the phase that appeared after cycling. Even if many factors may have an influence on the reduction from ^7^Li^+^ to metallic ^7^Li^0^, the diffusion and deposition of Li are probably decisive. Since the Li-rich phase appeared after the short circuit, it is likely connected to the formation of dendrites within the bulk of the sample, although without additional studies (e.g., 3D FIB-SIMS analysis), it is not possible to directly tie the surface formation of the Li-rich phase with bulk formation of Li-dendrites.

Given that our findings are based on a limited number of analyzed samples, interpretations should be treated with caution. The fact that only very low current densities were achievable in the setup shows the need for further optimization, both on the investigated components but also on the cell setup. However, this is the subject of ongoing work, whereas in this study the focus is on establishing a proof of concept of the *operando* workflow.

### Limitations

While *operando* analysis means analyzing “under operational conditions”, there are still different nuances to this definition. When designing an *operando* analysis, there are two ways of doing so: the first consists of simultaneous analysis and operation, which we propose to call “dynamic” *operando*. Another approach consists of operating a system and then pausing it at any given moment, thus freezing the system for a limited amount of time in this operational state to perform the analysis. By repeating this procedure multiple times at different stages of operation, we obtain data which might be defined as “static” *operando*.

Our proof-of-concept study classifies to the “static” mode. This has the drawback that when stopping the operation, the system seeks electrochemical neutrality and equilibrium, meaning that the system will not be in a perfectly frozen state. However, irreversible changes which occurr as a consequence of cycling, will be analyzable without the need for a perfectly frozen state. One advantage of the “static” mode is that the analysis and electrochemical cycling are decoupled, and therefore, the analysis has no influence on the actual operation itself. This is especially true for analyses using electrically charged particles such as ions (or electrons). For “dynamic” *operando* analyses, interferences between analysis and operation need to be considered during data interpretation.^45^

Since SIMS is a destructive characterization technique, it is important to evaluate the potential impact of the structural and chemical alterations on the electrochemical properties of the sample. For instance, it is known that the incorporation of Ga in LLZO can have an effect on conductivity.^46^ To investigate the range of these effects, SRIM^47^ (stopping and range of ions in matter) simulations were performed with parameters matching the experimental conditions. These results (details in Supporting Information under “2.1 Limitations”) elucidate that the sample depth impacted by the ion beam is ∼30 nm and that the maximum implanted Ga concentration is ∼3.7 at. % (Figure S5a). The maximal volume affected in this study is ∼30 × 30 μm^2^ × 0.03 μm = 27 μm^3^ per SIMS acquisition. For comparison, the volume of the LLZO pellet is ∼59.39 mm^3^, which is 9 orders of magnitude larger. Hence, it can be concluded that the volume affected by SIMS analysis as well as the Ga implantation is negligible, and therefore, the analysis does not significantly alter the overall sample structure or chemistry.

## Conclusions

We demonstrated in the present study the design as well as the proof of concept of an *operando* methodology for correlative structural, chemical, and electrochemical analysis. For this, we elaborated on the requirements of a dedicated sample holder and tested the final prototype with a variety of samples (commercial batteries, surface-mount resistors, ...). One technical challenge was to design a system without the need for major instrumental modifications. After troubleshooting and optimizing the sample preparation procedure, proof-of-concept measurements (see Figure 4) could be performed. The results show that the design is suitable for studying solid-state half-cells and has been successfully tested for “static” *operando* correlative structural, chemical, and electrochemical analysis. We were able to detect Li-rich dendrite phases after short-circuit failure and thereby could support previous studies by providing direct proof of Li through localized chemical analyses.

In this work, we especially focus on both extreme cases of a pristine state and a short-circuited state; however, our design allows the cycling to be stopped at any given moment to perform structural and chemical analysis and then to proceed with the cycling again. By analyzing multiple stages during cycling, the evolution of irreversible structural and chemical alterations can be studied. For this proof-of-concept study, it was important to force the battery to a point where structural and/or chemical alterations should become clearly visible, e.g., in a short-circuit.

Our method can also be used without any modifications to follow the evolution of battery degradation over many time steps. The *operando* methodology we present opens the doors to a variety of studies which aim to correlate electrochemical, structural, and chemical data of the exact same sample without the need to transfer between instruments. Furthermore, the implementation of SIMS in this workflow is extremely attractive for battery research as the instrument is capable of detecting and analyzing low-Z elements such as Li, which are difficult to analyze with conventional techniques, but are essential components of many batteries.

Apart from the *operando* capability of the custom-designed sample holder, there are additional features which make this design and the whole methodology attractive. As mentioned under “Design of the Operando Sample Holder”, the force of the springs (or rather the pressure which is applied to the sample) can be controlled and changed, which allows performing pressure-dependent studies and influencing especially the electrode-solid electrolyte interphase impedance, as seen in Figure 1b, or simply using consistent experimental conditions when analyzing a multitude of samples.

While this work has been performed with a solid-state half-cell, the sample holder design as well as the whole workflow are similarly useful for full battery cells. By adapting the pressure applied to the sample, solid polymer electrolytes as well as gel systems could be considered for this analysis. The set-up can be also useful beyond battery research. For example, the workflow could be considered for the analysis of photovoltaics,^48,49^ piezoelectric materials, or any materials which have pressure-dependent and/or electrochemistry-related processes.

We believe that by designing innovative and useful ways of analyzing batteries, we can accelerate the progress of battery technologies, which play a crucial role in the upcoming global energy transition.

## Supporting Information Available

Comparison (based on SEM, topography, and SIMS) between different sample preparation approaches; troubleshooting, testing, and validation measurements with commercial coin cells as well as resistors of the prototype operando sample holder; details about the IGTS for contamination-free transfer between the glovebox and the instrument; and SRIM simulation results to estimate the dimensions affected by the sputtering (PDF)
