# Supplementary material for: Toward Operando Structural, Chemical, and Electrochemical Analyses of Solid-State Batteries Using Correlative Secondary Ion Mass Spectrometry Imaging
Source: Anal Chem. 2023 Jun 22;95(26):9932–9. doi: 10.1021/acs.analchem.3c01059 (PMC10323867; doi:10.1021/acs.analchem.3c01059)
Supplement: Supplementary file 1 — ac3c01059_si_001.pdf [file ac3c01059_si_001.pdf]

Supporting Information

Towards *Operando* Structural, Chemical, and Electrochemical Analysis of Solid-State Batteries Using Correlative Secondary Ion Mass Spectrometry Imaging

Luca Cressa[a,b,*], Yanyan Sun[c], Dustin Andersen[a], Mathieu Gerard[a], Olivier De Castro[a], Dennis Kopljar[c], Maryam Nojabaee[c], Kaspar Andreas Friedrich[c,d], Guido Schmitz[b], Tom Wirtz[a], Santhana Eswara[a]

a Advanced Instrumentation for Nano-Analytics (AINA), Luxembourg Institute of Science and Technology, 41 rue du Brill, L-4422, Belvaux, Luxembourg

b Chair of Materials Physics, Institute for Materials Science, University of Stuttgart, Heisenbergstr. 3, 70569 Stuttgart, Germany.

c German Aerospace Center (DLR), Institute of Engineering Thermodynamics, Pfaffenwaldring 38-40, 70569 Stuttgart, Germany

d Institute of Building Energetics, Thermal Engineering and Energy Storage (IGTE), University of Stuttgart, Pfaffenwaldring 6, 70569, Stuttgart, Germany

*Corresponding author: E-mail: luca.cressa@list.lu

1. Materials & Methods

1.1 Sample preparation

1.2 Design and validation of the *operando* sample holder

1.2.1 Electrochemical experiments

1.2.2 Adaptation to inert gas transfer system and microscope

2. Results & Discussion

2.1 Limitations

Table

1. Materials & Methods

1.1. Sample preparation

To perform a proof of concept of the *operando* measurement, three different ways to divide the sample and treat the freshly exposed surface of interest were tested. As a plane sample surface is required to successfully perform SIMS analysis, we decided to divide the LLZO pellets in half. The three different preparation methods were:

- Cutting the LLZO pellet with a wire saw and cleaning with 1 M HCl followed by 1 h vacuum drying at room temperature.
- Cutting the LLZO pellet with a wire saw, polishing with SiC sandpaper up to #4000 and cleaning with 1M HCl followed by 1 h vacuum drying at room temperature.
- Physically breaking the LLZO pellet by hand and cleaning with 1M HCl followed by 1 h vacuum drying at room temperature.

In order to get a straight edge when physically breaking the LLZO pellet, a notch was carefully carved with a cutter knife to dictate the direction of breaking.

A comparison of the three sample preparation methods has been made based on the microstructural appearance (laser profilometer), the SEM image quality and the SIMS feasibility (Figure S1).

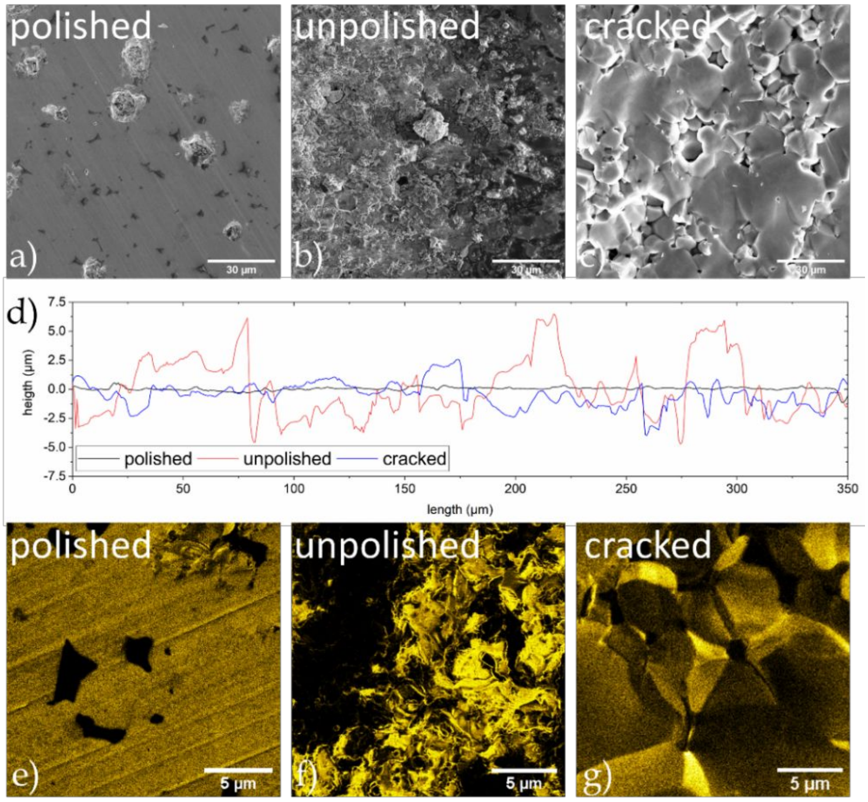

Figure S1: The top row shows SEM images of a) the polished, b) unpolished and c) cracked samples. The diagram in d) shows topographic line profiles of the three samples, which have been performed with a Keyence VK-X1100 Laser Scanning Confocal Microscope (LSCM). The black curve is representative for the polished sample, the red curve for the unpolished and the blue curve for the cracked sample. The last row (e, f and g) shows $^{139}La^{+}$ SIMS maps of the three samples. The cracked sample occasionally show relatively smooth microscale sections which are suitable for SIMS analysis.

Figure S1 a-c) show SEM images of a polished LLZO surface, an unpolished surface (cut via wire saw), and a sample surface which has been cracked by hand, respectively. The polished sample (Figure S1 a) has the advantage that it has the least topography, hence resulting in promising SIMS results. The unpolished sample (Figure S1 b) has a very rough topography with artefacts in SIMS. The line profiles in d) reveal that the polished sample is obviously the one with least topography. The unpolished sample shows topographic differences with up to 10 µm which is very unfavourable for SIMS analysis. The cracked sample reveals more pronounced roughness than the polished sample, however it has plateaus which are large and flat enough to successfully perform SIMS analysis. Unlike the polished sample, the cracked sample has the benefit that the granular shape of LLZO remains intact and that there is no cross-contamination possible related to cutting (often lubricant or liquid used) or polishing. One drawback of the polished sample is that polishing introduces small artefacts, e.g. lines that can clearly be seen in the SEM (Figure S1 a) and in the SIMS image (Figure S1 e). One the other hand, the cracked sample also presents a minor drawback: due to locally different incidence angles of the primary beam, slightly different sputter yields are obtained, resulting in different counts at different faces of a LLZO grain (details under **Results and Discussion**). Nonetheless, we decided that the cracked sample has the lowest risk of cross-contamination (structurally as well as chemically) and presents very good SIMS results in the ratio of sputter yields, hence was selected to be the sample preparation of choice for this proof-of-concept study.

1.2. Design and validation of the *operando* sample holder

1.2.1. Electrochemical experiments

The electrochemical experiments such as constant current cycling, chronopotentiometry or impedance spectroscopy were performed using a SP-150 potentiostat from BioLogic. All test measurements with commercial coin cells have been performed either outside or inside but vented FIB/SEM sample chamber to prevent contamination of the chamber due to possible outgassing. Several electrochemical tests have been performed with commercial coin cell batteries, to validate the prototype design and troubleshoot potential issues. Figure S2 a) shows an example of a chrono potentiometric discharge (with 20 mA from 3.2 V to ~1.5 V) of a RS Pro CR2032 3 V Lithium Manganese coin battery, 225 mAh (primary battery). The focus of this test was the optimisation of contact between micromanipulator and electrode. The black noisy line presents an example where the contact between the micromanipulator and the corresponding plate of the sample holder was not ideal. In this case, we contacted the electrode via the µ-tip (see SE image in Figure S1 b). To increase the contact area between micromanipulator and electrode, we decided to remove the µ-tip and instead to contact the electrode with the bigger supporting rod onto which the microscopic tip is usually attached. The red curve shows the same experiment but with an intimate contact between micromanipulator and electrode, resulting in a smooth discharge curve.

Figure S2 b) illustrates a SEM image showing the micromanipulator (i.e., supporting rod and the µ-tip). Figure S2 c) shows the experimental testing set-up outside the FIB-SEM instrument for testing commercial coin cells. Additional test measurements have been performed to validate and ensure a proper and artefact-free operation of our custom-designed *operando* sample holder. Figure S2 d) shows EIS test measurements which have been performed in a controlled atmosphere of a glovebox. The same Li/LLZO/Li sample has been measured in a conventional Swagelok cell and in the *operando* sample holder. The comparison of both shows that no considerable difference is noticeable, except that the impedance ($R_{SE/Li}$) is lower in the operando holder than in the Swagelok cell. Latter is attributed to different pressures being applied for clamping the sample.

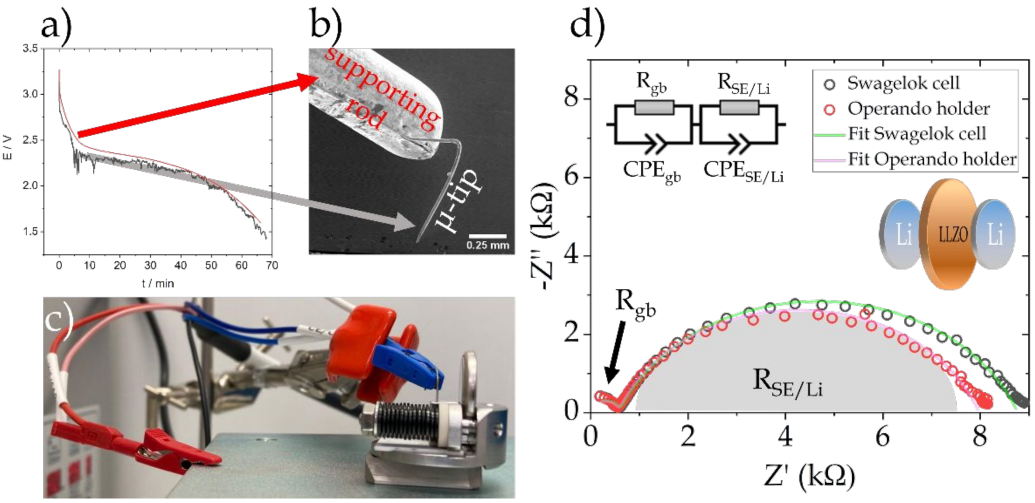

Figure S2: a) chronopotentiometry of a RS Pro CR2032 3 V Lithium Manganese coin battery with bad contact (black) and good contact (red) between micromanipulator and electrode. b) SEM image of the micromanipulator (supporting rod and the µ-tip) c) experimental testing set-up outside the FIB-SEM instrument. Red electrode attached to a lifting platform imitating the sample stage and the crocodile clip of the blue cable holding the micromanipulator that contacts the other electrode. d) EIS measurements of the same Li/LLZO/Li half-cell inside a conventional Swagelok cell and inside the operando sample holder. The equivalent circuit for the fitting is shown as inset and the impedance contributions of grain boundary (R_{gb}) and solid electrolyte - Li interface ($R_{SE/Li}$) are exemplarily shown.

Additionally, the electrical resistance owing to the experimental setup was assessed. These tests have been performed with two different surface mount resistors (100 Ω and 200 Ω), and for each the experiment has been performed with 1, 2 and 3 resistors. Figure S3 shows how the resistors were mounted on the sample holder and the zoom-in shows an SEM image of one of the resistors. The sample holder itself only adds an average of 1.3± 0.8 Ω to the system which is indeed negligible. The measurements performed with the sample holder inside the microscope and contacted using the micromanipulator add an average of 12.8 ± 3.5 Ω to the system, also this is negligible compared to the electrochemical systems which will be analysed.

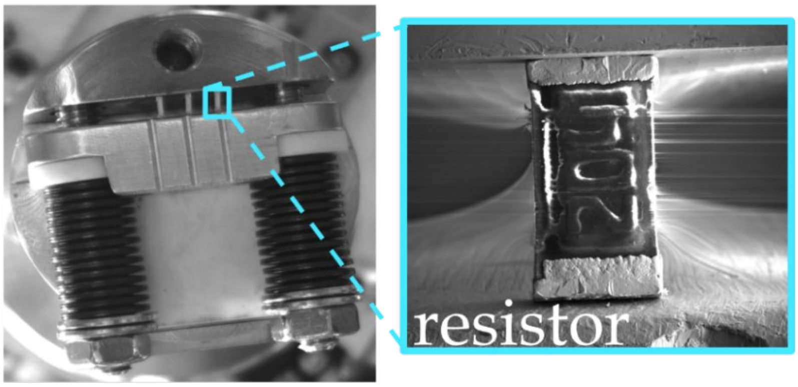

Figure S3: Operando sample holder with three resistors mounted. The zoom-in shows an SEM image of one of the resistors.

1.2.2. Adaptation to inert gas transfer system and microscope

A contamination free sample preparation and sample transfer is possible with an inert gas transfer system which in our case is composed of a transfer chamber on the microscope and a portable air- and vacuum-tight transfer box (Figure S4 a, b). This box can be introduced in a glove box, and the *operando* sample holder (with the sample) can be locked inside the transfer box. Subsequently the transfer box can be removed from the glove box and attached to the air lock at the microscope (Figure S4 c, d), where the argon atmosphere inside the box gets pumped before the sample can be introduced inside the microscope.

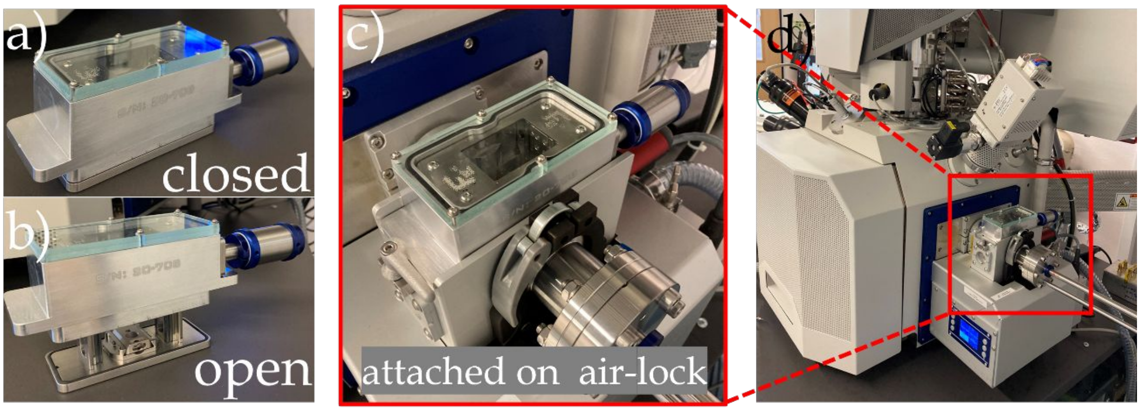

Figure S4: Inert gas transfer system and transfer box. a) closed and b) open transfer box c) transfer box mounted on the air lock, and d) a view of the transfer box, air lock and microscope.

2. Results & Discussion

2.1. Limitations

SRIM[1] (Stopping and Range of Ions in Matter) simulations have been performed to estimate the sample volume modified by the SIMS analysis. The simulation has been performed with 10^4 Ga ions with 30 keV energy and normal incidence angle (0°) impacting an amorphous LLZO target (5.1 g/cm^3). As seen in the simulation results shown in Figure S5 a), the maximum Ga concentration (~3.7 at.%) appears at a depth of approximately 20 nm, additionally the maximum number of vacancies (~0.2 per Ga ion) occurs at a depth of 10 nm. The mean longitudinal range of the Ga ions is 18.2 nm however the full interaction volume expands until approximately 40 nm. The fraction of ions reaching a depth beyond 30 nm is so small (<1 at.%) that its effect can be neglected. Figure S5 b) shows a 2D representation of primary ion trajectories (Ga^+) inside the LLZO phase. Both axes represent distances (in nm), the arrow shows the location of initial impact, and the red lines represent individual trajectories of 10^4 Ga-ions travelling through LLZO. These simulations have been performed to estimate the depth (and volume) which is affected by SIMS analysis.

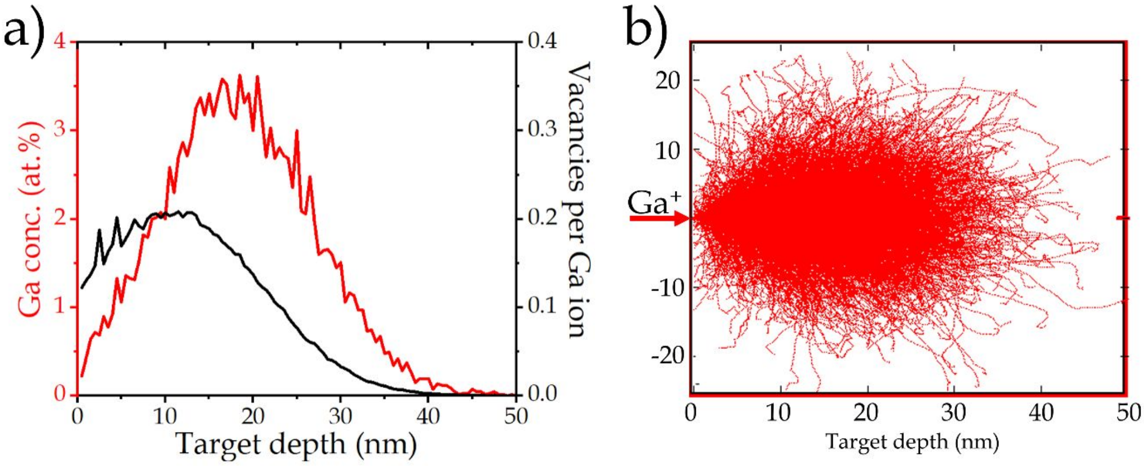

Figure S5: a) Concentration profile of implanted Ga (red) and number of created vacancies per Ga ion (black). b) Primary ion trajectories (Ga^+) in an amorphous LLZO phase (density 5.1 g/cm^3). Both axes represent distances, the arrow shows the location of ion impact and the red lines in the diagram represent individual trajectories of 10^4 Ga-ions travelling inside the LLZO phase.

Table

Table S1: Fit parameters for impedance spectroscopy of a symmetric Li/LLZO/Li cell shown in Figure 1b). R represents an ohmic resistor and CPE a constant phase element. The indices are gb: grain boundary, SE/Li: Solid electrolytes/Li-electrode and u: unidentified. The exponent for the CPE is denoted as n.

name	value	fit error	unit
R_{gb}	707.733	16.201	Ω
CPE_{gb}	1.32E-09	2.65E-10	$F \cdot s^{(n-1)}$
n_{gb}	0.899	0.013	/
$R_{SE/Li}$	14978.706	2030.624	Ω
$CPE_{SE/Li}$	4.07E-08	3.57E-09	$F \cdot s^{(n-1)}$
$n_{SE/Li}$	0.860	0.019	/
R_u	9146.892	2100.510	Ω
CPE_u	1.08E-06	2.91E-07	$F \cdot s^{(n-1)}$
n_u	0.702	0.035	/

The EIS measurements (Figure 1 b) were done in potentiostatic mode in a frequency range from 1 MHz to 1 Hz with an amplitude of 5 mV. The bulk resistance is not accessible in the frequency range which was used for this experiment. However, a third semi-circle (impedance at low frequencies) of unidentified origin is needed to fit the experimental data. A detailed investigation of this is beyond the scope of this study.

References

(1) Ziegler, J. F.; Ziegler, M. D.; Biersack, J. P. *SRIM – The stopping and range of ions in matter (2010). Nuclear Instruments and Methods in Physics Research Section B: Beam Interactions with Materials and Atoms* **2010**, *268*, 1818–1823.
